# Assessment of the intrasinusidal volume before and after maxillary sinus augmentation using mri – a pilot study of eight patients

**DOI:** 10.1186/s12903-024-03858-x

**Published:** 2024-01-29

**Authors:** K. Flick, R. Smeets, M. Gosau, L. Meyer, U. Hanning, A. A. Kyselyova, C. Scheifele, B. Höhmann, A. Henningsen

**Affiliations:** 1 Department of Oral and Maxillofacial Surgery, University Medical Center Hamburg-Eppendorf, Martinistrasse 52, 20246 Hamburg, Germany; 2 Division of “Regenerative Orofacial Medicine”, Department of Oral and Maxillofacial Surgery, University Medical Center Hamburg-Eppendorf, Hamburg, Germany; 3 Department of Diagnostic and Interventional Neuroradiology, University Medical Center Hamburg-Eppendorf, Hamburg, Germany; 4 Dental Radiology Unit, Center for Dental and Oral Medicine, University Medical Center Hamburg-Eppendorf, Hamburg, Germany; 5 Department of Preventive and Restorative Dentistry, Center for Dental and Oral Medicine, University Medical Center Hamburg-Eppendorf, Hamburg, Germany

**Keywords:** Magnetic resonance imaging, Sinus floor augmentation, Maxillary sinus graft, Dental implants, 3 T, MRI

## Abstract

**Purpose:**

The purpose of this study was to evaluate the suitability, accuracy, and reliability of a non-invasive 3-Tesla magnetic resonance imaging technique (3 T-MRI) for the visualization of maxillary sinus grafts in comparison to conventional, X-ray-based, established standard imaging techniques.

**Methods:**

A total of eight patients with alveolar bone atrophy who required surgical sinus floor augmentation in the course of dental implantation were included in this pilot study. Alongside pre-operative cone-beam computed tomography (CBCT), 3 T-MRI was performed before and 6 months after sinus floor augmentation. Two investigators measured the maxillary sinus volume preoperatively and after bone augmentation.

**Results:**

In all cases, MRI demonstrated accurately the volumes of the maxillary sinus grafts. Following surgery, the bony structures suitable for an implant placement increased at an average of 4.89 cm^3^, corresponding with the decrease of the intrasinusidal volumes. In general, interexaminer discrepancies were low and without statistical significance.

**Conclusion:**

In this preliminary study, we could demonstrate the feasibility of MRI bone volume measurement as a radiation-free alternative with comparable accuracy to CT/CBCT before procedures like sinus floor augmentation. Nevertheless, costs and artifacts, also present in MRI, have to be taken into account. Larger studies will be necessary to justify the practicability of MRI bone volume evaluation.

**Supplementary Information:**

The online version contains supplementary material available at 10.1186/s12903-024-03858-x.

## Background

The lateral sinus floor augmentation was first introduced by Tatum in 1976 and later on published by Boyne and James in 1980, who also proposed the additional use of biomaterials in order to elevate the Schneiderian membrane [1–3]]. In 1994 Summers proposed a technique for immediate implant placement using a crestal approach that is today known as an internal sinus floor augmentation [3]. These publications led to a groundbreaking development for modern implantology.

Established imaging modalities such as panoramic tomography, tooth radiographs, cone-beam computed tomography (CBCT) or computed tomography (CT) for preoperative planning and postoperative control of dental implants or bone augmentations are based on X-ray imaging and carry potential risks for patients. CBCT is currently regarded the gold standard in dental implant planning [4–6]. However it may lack sufficient soft tissue contrast which can be a disadvantage [7].

In contrast, MRI (magnet resonance imaging) represents a non-radiographic imaging alternative for which there are very few documented short and long-term side effects for this specific application area in dentistry [8, 9]. For example, Khan et al. carried out an experimental study in mice using a high field MRI to study the mercury release from amalgam fillings and the associated side effects. They found an only transient short-term impairment in terms of balance and locomotor activity and concluded that the short exposures to high-field static magnetic field (SMF) up to 23.0 T have negligible side effects on the health [10]. Aside from potential physical consequences, MRI is often associated with high levels of anxiety during the examination [11]. In the past, many of the studies dealing with MR imaging of the maxillary sinus or dental regions in general were performed in the form of ex vivo or animal studies. For example, in 2014, Korn et al. placed PEEK implants in three mini-pigs and performed MRI and histomorphologic studies postoperatively [12]. In 2020, Flügge et al. studied 16 human bisected mandibles using MRI in vitro and found that all relevant anatomical structures for imaging diagnostics in implant dentistry could be displayed with MRI. The study showed that the accuracy of MRI-based fully guided implant placement in vitro was comparable to the workflow using CBCT [13].

However, a 2016 systematic review by Niraj et al. already concluded that MRI can be used for implant diagnosis and treatment planning [14]. Recent experimental in vivo studies provided even more promising data: Laurino et al. studied 15 patients and Flügge et al. studied 10 patients in vivo, both with equally satisfactory results [4, 6]. Flügge et al. used an intraoral coil on 10 patients pre- and postoperatively and found that MRI appears to be eligible for the display and longitudinal observation of autologous onlay bone grafts [6]. Laurino et al. studied the correlation between CBCT and MRI regarding the sinus graft heights in different weightings in 15 patients and found a strong correlation between CBCT and MRI measurements and that both tested sequences may be used to for sinus graft assessments. The correlation between T2-weighted MRI sequences and CBCT was slightly higher than between T1-weighted MRI sequences [4].

However, in the most recent systematic review, which included 10 studies, Fuglsig et al. concluded that more studies on the accuracy of MRI are needed to establish it as a suitable imaging modality to replace CT and CBCT [15]. In another scientific review from 2021, Reda et al. highlighted the potential of MRI for diagnosis in dental practice [16]. With the technical progress in MRI diagnostics - for example the 3-Tesla technology - new imaging possibilities arise, especially for the visualization of hard tissue structures. Therefore, re-evaluating MRI as a non-radiological alternative for pre-surgical and implant imaging possibilities appears reasonable and necessary [13, 17–19].

Considering that MRI could be regarded as an alternative to conventional imaging protocols in dental implants, it should also be noted that additional soft and hard tissue findings, such as temporomandibular joint dysfunction, periodontitis, chronic sinusitis, detection of odontogenic inflammation, caries, peri-implant disease, and visualization of root canals are possible with MRI [20–22]. Additionally, the degree and progression of bone graft mineralization might be assessable. Widek et al. assessed the mineralization of third molars using MRI for the purpose of age estimation. In summary, the authors concluded that dental MRI holds promise as an alternative to conventional panoramic radiograph based age assessment [23]. In a different study, Bohner et al. visualized the trabecular bone morphology in porcine bone samples using MRI. They concluded that within the limitations of the study, MRI overestimated trabecular-bone parameters, but with a statistically significant fixed linear offset and called for further studies to determine the clinical feasibility of MRI for trabecular-bone assessment [24].

The potential long-term benefit to the general public is now to determine the suitability of MRI for dental issues in order to avoid radiation exposure from X-ray examinations in the future [25]. MRI technique has evolved considerably over the years. Originally, it was only used up to the neuroaxis, but more recently it has been extended to all parts of the body, including the oral cavity, either alone or in combination with other techniques to achieve the highest diagnostic accuracy. The rapid growth in clinical applications has been accompanied by numerous technologic advances in MR imaging over the past few years [14].

The purpose of this study was to evaluate the suitability, accuracy, and reliability of a non-invasive 3-Tesla magnetic resonance imaging technique for visualization of maxillary sinus grafts in comparison to conventional X-ray based established standard imaging techniques in 9 patients. The hypothesis was, that MRI can be used for preoperative planning and postoperative controls in sinus lifting procedures.

## Methods

### Patient selection: inclusion and exclusion criteria

The study protocol of this clinical, non-randomized, controlled, prospective study with a small but reliable number of patients was approved by the Ethics Committee of the Hamburg Medical Association with the approval number “PV 5784”. Written informed consent for imaging was obtained from each participating patient. The inclusion criteria were that patients had to be over 18 years of age and required a surgical-prosthetic implant solution in the maxilla for a single-tooth gap or free-end situation.

Exclusion criteria were: previously irradiated bone, severe systemic diseases (e.g., uncontrolled diabetes mellitus), haemorrhagic diathesis, heavy smokers (> 15 cigarettes per day), bisphosphonate therapy, immunodeficiency, poor compliance, presence of metallic foreign bodies (including vascular clips or piercings), patients with non-MRI-compatible pacemakers, defibrillators, cochlear implants, patients with known fear of small spaces (claustrophobia), patients with non-MRI-compatible implanted insulin pumps or neurostimulators, and pregnant women. However, no patient was excluded from the study on the basis of these criteria.

Statistical sample size planning, assuming a range of equality of measurements between CBCT and MRI of 0.1 mm and a standard deviation of 0.1 mm, yielded a power of 81.5% (α = 0.05; β = 0.19) in 8 patients. Therefore, a sample size of 8 patients was considered representative. To minimize the risk of statistical interferences due to possible drop-outs, 9 patients were included.

A total of 9 patients received sinus floor augmentation as part of their dental implant treatment at the University Medical Center Hamburg-Eppendorf (UKE). The patients were acquired from the Department of Oral and Maxillofacial Surgery and the Department of Periodontology, Preventive and Restorative Dentistry at the University Medical Center Hamburg-Eppendorf (UKE).

### Methods

The clinical routine in this study included a preoperative CBCT (Cone beam computed tomography, Planmeca Oy Viso G7, Asentajankatu 6, FIN-00880 Helsinki, Finland) for dental implant planning and assessment of the initial anatomical situation and the residual bone height.

Additionally, to the clinical routine, a preoperative 3 T MRI examination (T0) and another MRI 6 months postoperatively (T1) were performed to analyse the osseointegration of the graft and the newly created vertical bone height and bone volume of the maxilla.

### Surgical treatment

Nine patients underwent maxillary bone augmentation for implant placement, ranging from single tooth gaps to complex bone block grafting in patients with cleft palates. Both unilateral and bilateral procedures were performed. Seven patients received an external sinus floor augmentation and 1 patient received an internal sinus floor augmentation. In 8 cases, patients were treated with a combination of BioOss (Geistlich Pharma AG, Switzerland) and autologous bone. In addition to the graft material, 3 of the bone mixtures were enhanced with autologous PRGF. Simultaneous implant placement was performed in 7 patients, 2 patients were treated using a two-step approach.

Surgical procedures were performed under local anesthesia using Ultracain D-S forte, (Sanofi, Paris, France). A crestal incision was made with medial and distal relief into the vestibulum and a visualization of the facial wall of the maxillary sinus was performed. In cases of external sinus floor elevation, a sinusoidal window was created using rose drill, piezosurgery (Mectron s.p.a., Carasco, Italy) or safescrapers (Zantomed GmbH, Duisburg, Germany) and the Schneiderian membrane was displayed. Preparation with hand instruments to mobilize the sinus membrane was carried out and the integrity of the membrane checked. The augmentation of the maxillary sinus was carried out using autologous bone (50%) and bone substitution material (50%) (BioOss, Geistlich Pharma AG, Switzerland). In 3 cases a membrane was used to cover the sinus window membrane (BioGide membrane, (Geistlich Pharma AG, Switzerland) or a collagene membrane (Bego GmbH& co. KG, Bremen, Germany). The surgical approach was closed using Vicryl 5.0 (Ethicon Johnson & Johnson Inc., New Brunswick, USA) and Seralon 4.0 (Serag-Weissner GmbH & co. KG, Naila, Germany).

In the case of the internal sinus floor augmentation a crestal incision was made and subperiosteal exposure with the raspatory was performed. Exposure of the bone and curettage for complete removal of all soft tissue was carried out. Subsequently the preparation of the implant site according to the drilling protocol and an internal sinus lift using osteotomes was performed. No perforation of the Schneiderian membrane could be detected. From occlusally, a mixture of 50% autologous bone and 50% bone substitute material (BioOss, Geistlich Pharma AG, Switzerland) was inserted and implant was placed using a ratchet. The closure screw was inserted using CHX gel. The surgical approach was closed with multiple layers using Vicryl 5.0 (Ethicon Johnson & Johnson Inc., New Brunswick, USA) and Seralon 4.0 (Serag-Weissner GmbH & co. KG, Naila, Germany).

A postoperative X-Ray-based orthopantomography was performed (Planmeca, ProMax 2D S3, Helsinki, Finland).

### MRI data evaluation

MRI scans were performed with a whole-body 3 T MRI scanner (Siemens Prisma, Siemens Healthineers GmbH, Erlangen Germany) preoperatively and 6 months postoperatively in the Department of Diagnostic and Interventional Neuroradiology at the University Medical Center Hamburg-Eppendorf. The standardized MRI protocol included: fat-suppressed proton density space with multiplanar reformations, isotropic T1 weighted VIBE (volumetric interpolated brain examination) with multiplanar reformations, axial fat-suppressed T2 weighted space, axial T1 weighted PETRA (pointwise encoding time reduction with radial acquisition) and axial T2 weighted HASTE (half-Fourier acquisition single-shot turbo spin-echo) sequences. Layer thickness was 0.5 mm.

Two investigators with over 5 years of experience in diagnostic neuroradiology independently measured the bone volume of the maxillary graft, respectively the reduction of air in the maxillary sinus and recorded the volume changes over time using area measurement tool using the special syngo.via software (Siemens Healthineers Siemens GmbH, Erlangen, Germany) on a RaDiForce MS230W monitor (Eizo, Hakusan Ishikawa, Japan).

**Fig. 1:**
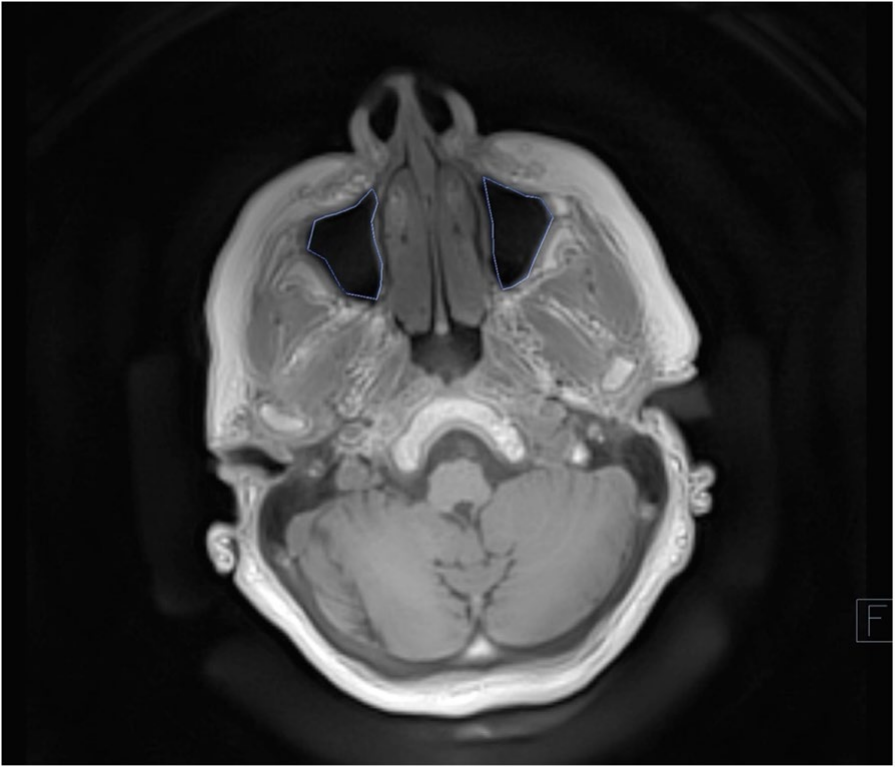
Preoperative MRI scan: marking of the outline of the maxillary sinus, transversal layer, T1 weighted petra sequence

**Fig. 2:**
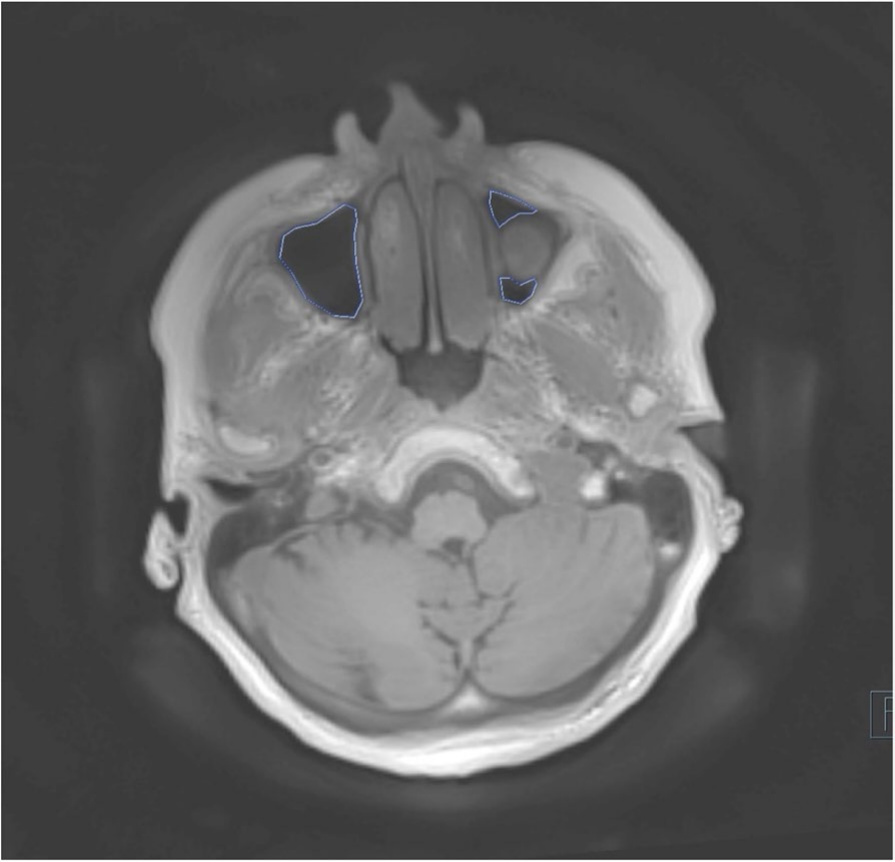
MRI, 6 months postoperatively, marking of the sinus with cut out of the bone graft, transversal layer, T1 weighted petra sequence

**Fig. 3:**
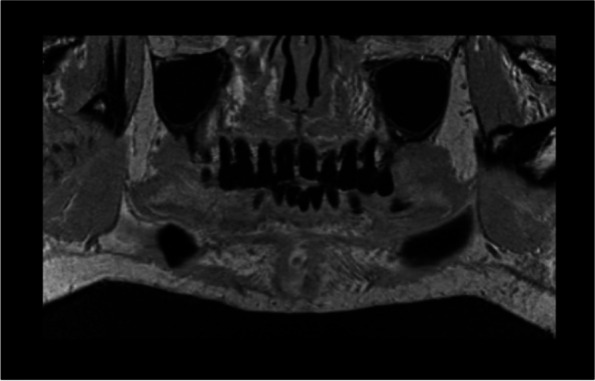
Preoperative MRI before vertical bone augmentation, coronal T1 weighted showing no pathological findings

**Fig. 4:**
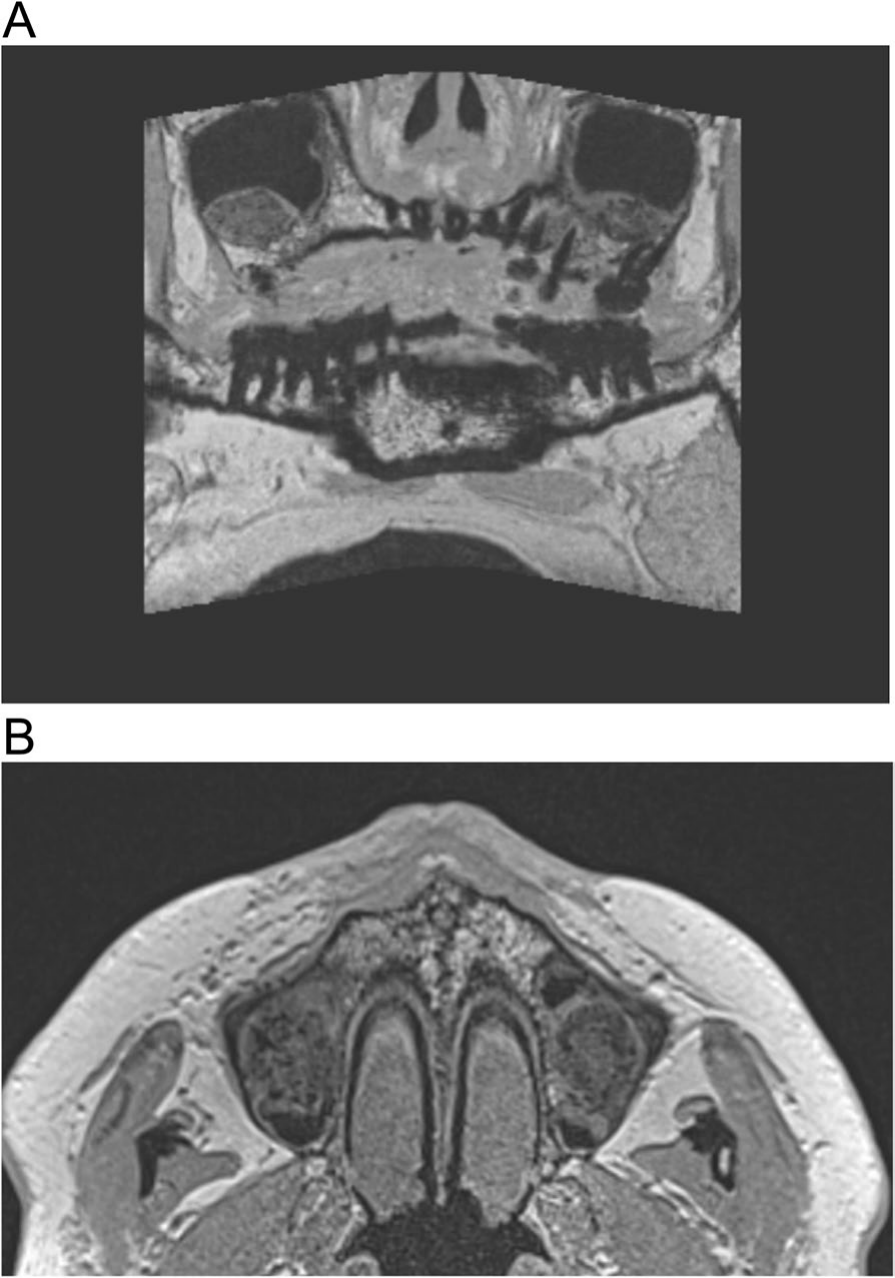
**A** MRI, 6 months postoperatively, coronal layer, T1 weighted. **B** MRI, 6 months postoperatively, transversal layer, T1 weighted sequence

**Fig. 5:**
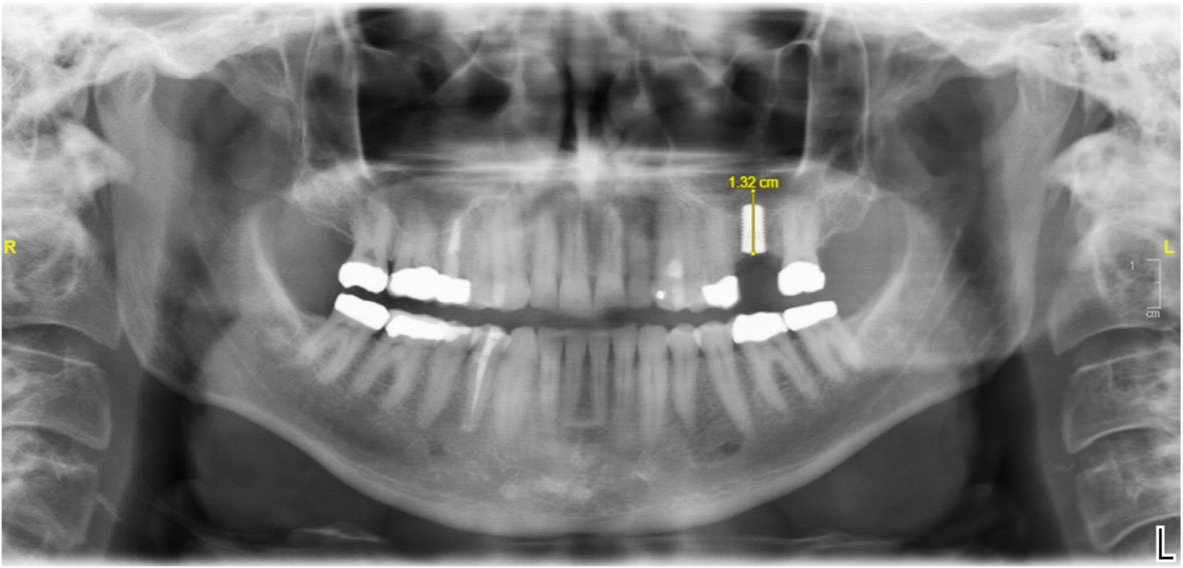
Panoramic image 6 months postoperatively showing a 10 mm implant and measurement of the augmented bone at the highest point

The investigators manually sketched the borders of the maxillary sinus in each layer on the T1 weighted transversal petra sequence to calculate the volume of the maxillary sinus preoperatively. The new extent of the maxillary sinus in each layer was redrawn after 6 months of healing, omitting the bone graft, resulting in a reduction of the maxillary sinus (Figs. 1, 2, 3, 4 and 5). On examination, the mucosa and the newly formed bone showed different signal patterns on the T2 space fat-saturated images, making it possible to distinguish between the two tissues.

### Panoramic image evaluation

To verify the diagnostic procedure, the inter-rater reliability was determined by measuring the implant length and the augmented bone height by two neuroradiological experts. The panoramic tomogram served as the basis for the measurement (Table 1).

**Table 1:** Measured bone height from panoramic tomography

Patient number/implant number	Measured Bone height in mm	Measured Bone height in mm
*1.1*	*2,6*	*3,2*
*1.2*	*3,9*	*3,4*
*1.3*	*4,5*	*4,7*
*2.1*	*3,2*	*4,1*
*3.1*	*2,7*	*2,6*
*4.1*	*5,5*	*4,7*
*4.2*	*2,2*	*1,5*
*4.3*	*4,4*	*4*
*4.4*	*7,0*	*7,2*
*5.1*	*3,6*	*3,8*
*5.2*	*5,5*	*5*
*5.3*	*5,2*	*5,4*
*5.4*	*2,4*	*1,2*
*6.1*	*4,8*	*3,8*
*6.2*	*3,5*	*3,4*
*6.3*	*3,6*	*3,7*
*7.1*	*1,8*	*1,5*
*7.2*	*1,6*	*2*
*7.3*	*2,0*	*3*
*7.4*	*1,4*	*2,5*
*8*	*2,7*	*3,2*

Rater 1: average 3,53 +/− 1,5

Rater 2 average 3,52 +/− 1,4

### Patient questionnaire

All patients were given a questionnaire after each examination with the purpose to evaluate the potential individual additional stress caused by the MRI examination. The aim of the patient questionnaire was to determine the possible physical symptoms of the patients as well as the psychological stress during the MRI examination. Patients could indicate the intensity of each sensation on a scale of 1 to 4 (Appendix 1). The questionnaire included a section to be completed only after the second MRI. Its purpose was to assess the change in perception when patients received more than one MRI examination. The methodology of this questionnaire was based and modified on the study of Modolo et al. published in 2017 [26].

### Statistical analysis

IBM SPSS statistical software version 27 was used as the basis for data processing and analysis (IBM, version 27.1).

The sample size calculation was performed with Proc Power of the SAS software version 9.4. A value of 0.1 was taken as the mean difference and the standard deviation of the difference can also be estimated at 0.1. The intra-individual correlation is conservatively estimated at 0.65, but is probably closer to > 0.75. The power should be at least 0.8. The calculation is performed with a two-sided PAIREDMEANS statement.

All available data, including the results of the questionnaire, were analyzed descriptively. The structure of the data itself had a paired design due to the before and after comparison, indicating a paired t-test for primary analysis. Since the ratings of two raters were available from each observation time point, mean values were calculated from both ratings for primary analysis.

Due to the metric structure of the discordances of the two rates, the Pearson correlation coefficient was used to calculate the inter-rater reliability.

A sensitivity analysis was used to confirm no confounding effects regarding the different sides of the jaws. Here, further confounders (such as the side of the jaw) were included in the statistical model within the context of linear regression in order to exclude a clinically relevant influence. The statistical comparison of the averaged ratings was performed using a paired t-test. *P*-values of *p* < 0.05 were considered to be statistically significant.

## Results

### Patient data

Patients who participated in this study were between 36 and 75 years of age at the time of the survey. Four patients were female and 5 patients were male. Five patients underwent bilateral sinus floor augmentation surgery. Thus, a total of 12 sinus elevation procedures could be assessed (Table 2).

**Table 2:** Patient data

Patient Number	Age	Bone graft region	Gender	Sinus floor augmentation
*1*	*50*	*15–16, 24–27*	*female*	*bilateral*
*2*	*49*	*15–16, 25–26*	*male*	*bilateral*
*3*	*48*	*25*	*male*	*unilateral*
*4*	*75*	*16, 26–27*	*female*	*bilateral*
*5*	*64*	*26*	*female*	*unilateral*
*6*	*58*	*15*	*male*	*unilateral*
*7*	*52*	*15–17*	*male*	*bilateral*
*8*	*63*	*16–17, 25–27*	*male*	*bilateral*
*9*	*36*	*16*	*female*	*unilateral*

Due to a strong claustrophobic feeling, one patient dropped out of the study at the first examination

### Bone volume reconstructed from MR imaging

The calculated volume of the augmented bone 6 months postoperatively ranged from 2.1 to 7.7 cm^3^ and was in mean 4.89 ± 4.43 cm^3^ (Table 3).

**Table 3:** Calculated bone volume: T0 - calculated preoperative maxillary sinus volume, T1 - postoperatively reduced volume of the sinus

	Volume of the maxillary sinus in cm^3^	Standard deviation in cm^3^
*T0*	*19.26*	*6.36*
*T1*	*14.46*	*6.74*

The interclass correlation as a measure for the interval-scaled reliability was ICC = 0.86.

#### Bone height measured from postoperative panoramic tomography

## Questionnaire

Analysis of the questionnaire revealed that the vast majority of patients reported no discomfort or additional distress from the MRI protocol. Only one of the nine study patients experienced a state of claustrophobia immediately upon entering the MRI. This caused one patient to drop out. However, the patient’s discomfort and claustrophobic symptoms disappeared immediately after interruption of the MRI examination, so it can be assumed that no long-term consequences are to be expected in this patient. Since this patient dropped out of the examination, they only completed the first half of the questionnaire. They indicated “strong anxiety” and “strong discomfort” during the examination and “no concerns at all” before the examination. General discomfort was indicated as “strong discomfort.” (Tables 4 and 5).

**Table 4:** Questionnaire analysis ranging from 1 to 4: 1 = no concern, no fear, no pain, no discomfort at all, 4 = heavily concerned, strong fear, very painful, strong discomfort

Indication of concern before the examination	Indication of fear during the examination	Indication of pain during the examination	Indication of general discomfort
9 patients voted “no concern at all”	8 patients voted “no fear at all”	9 patients voted “no pain at all”	7 Patients voted “no discomfort at all”
	1 patient voted “strong fear”		1 patient voted “slight discomfort”
			1 patient voted “strong discomfort”

**Table 5:** Questionnaire analysis ranging from 1 to 4:1 = no concern, no fear, no pain, no discomfort at all, 4 = heavily concerned, strong fear, very painful, strong discomfort

Indication of concern before the examination	Indication of fear during the examination	Indication of pain during the examination	Indication of general discomfort	Change of perception between the two examinations
8 patients voted “no concern at all”	8 patients voted “no fear at all”	8 patients voted “no pain at all”	7 Patients voted “no discomfort at all”	8 Patients voted “no change in perception”
			1 patient voted “slight discomfort”	

### Evaluation after the first MRI

#### Evaluation after second MRI

### Accuracy of MR imaging

The accuracy of the MRI concerning the implant site was so exact that it could have served as a basis for implant planning. In addition, the image contrast was excellent and the graft volumes could be assessed without complications or limitations. In summary, the accuracy can be considered very satisfactory, Magnetic artefacts had no significant influence on the quality of the MRI imaging or the ability to access the pre- and postoperative volumes of the maxillary sinuses. Thus, the initial hypothesis is to be accepted.

### Additional findings in MR imaging

In the present exemplary study, no additional findings such as chronic sinusitis, bone vitality, and impaired vascular status were collected. The MRI protocol also allowed assessment of soft tissue components as well as the temporomandibular joint showing no pathologies. Additionally, no pathologies regarding bony structures were found. Preoperative knowledge of the exact position and location of the inferior alveolar nerve is also of great surgical relevance, as it would help preventively to avoid potential injuries. In this study, it was possible to locate and track the alveolar nerve on the MR images and to measure the vertical bone height of the mandible over the nerve (Appendix 2). In a 2021 systematic review that included 30 studies that addressed imaging of the alveolar and lingual nerves using MRI, it was concluded that MRI is a promising imaging modality that could become part of routine clinical practice [27].

### Statistical results

In the analysis, the mean volume (averaged over the two rates and all patients) for the period before augmentation was 19.36 cm^3^ with a standard deviation (SD) of 6.63. Six months after surgery, a reduced mean volume of 14.46 cm^3^ (SD = 6.74) was observed. Consequently, there was a mean reduction of − 4.89 cm^3^ with a standard deviation of 4.42 and a 95% confidence interval of [− 7.71; − 2.79]. The reduction can be considered statistically significant with t(11) = − 3.827 and *p* = 0.003. This results in a standardized effect size of Cohen’s d = − 1.105.

The inter-rater correlation according to Pearson yielded a coefficient of ρ = 0.765.

Sensitivity analyses revealed no other discernible confounders within the scope of the analytics.

## Discussion

### Key results

MRI provides a non-radiographic alternative in medical imaging [7]. With the introduction of 3 T MRI technology, the level of detail has increased significantly and it may now be possible to answer specific dental and implantological questions [28]. This study aimed to evaluate the suitability, accuracy and reliability of 3 T magnetic resonance imaging for visualization of maxillary sinus grafts. The calculated volume of the augmented bone 6 months postoperatively ranged from 2.1 to 7.7 cm^3^ and was in mean 4.89 ± 4.43 cm^3^ (Table 3). The image contrast was excellent and the graft volumes could be assessed without complications.

However, there are some limitations and disadvantages which cannot be ignored.

## Limitations

MRI is still associated with high costs, as the acquisition and operating costs for MRI are significantly higher than for X-ray-based procedures. In addition, MRI has a significantly longer examination time than X-ray-based examinations and is less accessible to oral surgeons than established imaging examination methods [14]. On average the examination time was 30 minutes per patient.

The longer examination times of MRI compared with CT or conventional radiographic examinations may be perceived as uncomfortable by patients. MRI is not possible in case of claustrophobic patients. Most of patients did not suffer from anxiety in the narrow MRI tube, but it still needs to be considered that only 8 patients could be included and one patient in this study dropped out.

Additionally, MRI has certain limits with metallic foreign bodies. Although MRI, like CBCT, is susceptible to artefacts that may be caused by metal objects or patient motion, the general contraindications such as metallic foreign bodies, pacemakers, cochlear implants, etc., are an absolute limitation to the general use of MRI [29]. This study also revealed severe artefacts caused by metallic or ceramic restorations in the occlusal plane. However, they had no influence on the exact assessment of the augmented bone areas. The exclusions included patients with non-MRI-compatible pacemakers, defibrillators, cochlear implants, insulin pumps or neurostimulators. Even if no patients were excluded in this study for these reasons, it is possible that this could limit the applicability of the findings in a clinical setting. However, it should be noted that the number of patients with non-MRI-capable devices is now very low and that the use of MRIs is no problem with newer generations of devices.

A further limitation of this study is the small number of patients (8 patients) and the large differences in the augmented bone volumes, which resulted in high standard deviations.

## Interpretation

The calculation of the bone volumes was possible in all cases without any problems, as likewise described by Flügge et al. in 2020. These authors also assessed the bone volume by manual segmentation without difficulties [6]. The special feature of these studies was the measurement of the bone volume by segmentation and not only the two-dimensional display of a bone height as with other authors. In the present study, two-dimensional bone height was measured in addition to bone volume. The measurement results regarding bone height did not differ significantly between the researchers, also because the measurements were uncomplicated and unambiguous. In this study, artifacts did not affect the interpretation of MRI images. However, artifacts present differently in MRI and may be problematic in imaging and measuring depending on both the implant material, for example titanium or zirconia, as well as the particular weighting in MRI [5, 30–33]. In addition, the composition of the individual intraoral restorations can strongly influence the artifact dimension [34, 35]. Systematic reviews have confirmed that artifacts fundamentally depend on the materials used, the location in the oral cavity, and magnetic resonance parameters [36]. It should also be considered that titanium implants are causing artifacts in conventional imaging protocols as well [37]. Some authors even suggest that MRI is superior to CT regarding implant planning and artifacts [38]. In this study, artifacts caused by metal foreign bodies were very low and did not represent a disadvantage in the measurement of maxillary sinus dimensions. In conclusion, there are no available imaging techniques that are not susceptible to artifacts at all. Recent studies have shown that certain sequences can reduce artifacts even further [39].

In contrast to certain limitations with artifacts, the addressed clear benefits of MRI in relation to the visualisation of hard and soft tissue structures may become additionally helpful to improve the proper placement, durability and implant survival. In accordance with the current literature, this study demonstrated that the measurement of the maxillary sinus was accurate, reliable, and unproblematic for each scan.

Generally, highly elective procedures such as sinus floor augmentations associated with dental implants would not be performed during pregnancy, but it is another benefit not yet addressed that should be mentioned that MRI can also be used for pregnant women and children and even for indications other than implants. Even though in this experimental study pregnant women and children were excluded, MRI examinations in general would be possible and are regarded as safe [14].

In this study, radiation-free MRI provided highly detailed images of the relevant soft tissues and allowed clear differentiation of tissue types, which could, for example, facilitate the detection of irregularities or inflammatory processes. The ability to image structures such as the alveolar nerve and the lingual nerve could not only become a helpful tool for preoperative checks, but also be part of clinical follow-up care [27].

Bennardo et al. published a retrospective single centre study in 2022 removing implants that where displaced in the maxillary sinus. They performed a preoperative computed tomography on all surgical candidates and concluded that a transnasal or transoral approach or a combination of both can be used safely in cases of implant displacement or migration in the maxillary sinus [40]. It would be conceivable, that even in these cases radiation intensive computed tomography could potentially be replaced by radiation free MRI. Although X-ray-based techniques, such as CT or conventional X-ray are known to be better for examining bones, this study also showed excellent results in imaging bone structures, leading to the assumption that 3 T-MRI may partially replace X-ray-based techniques in the future to avoid X-ray radiation, especially in X-ray-sensitive patients such as children and pregnant women. This is also consistent with current systematic reviews in the existing literature [9, 21]. In this study, measurement of the vertical bone height on the conventional panoramic images was possible without any problems, but unlike MRI, no volume could be measured on the postoperative radiographs, thus missing an evident piece of information. As this study has provided further evidence of the reliability of MRI in the measurement of bony structures and the differentiation between hard and soft tissue, its further use in in vivo research is also supported [41].

Critically, the data were obtained by manually measuring the extent of the maxillary sinus before and after surgical augmentation of the maxillary sinus floor, and no algorithm-based representation of the maxillary sinus was possible. As with any manual measurement, they are prone to error by the individual researcher when measuring the maxillary sinus in each slice. However, the susceptibility to error may be further minimized in the future by increasing technological development, not least the accelerated development of artificial intelligence, which is becoming increasingly important [42].

Finally, the time and cost disadvantages could be overcome if the medical device industry were given sufficient incentive to develop smaller devices for dental use that would allow shorter examination times and thus lower financial expenditures.

## Conclusion

3 T-MRI appears to be a generally suitable and promising non-radiant imaging technique for pre-prosthetic and implant surgery. It shows excellent image contrast not only for solid but also for soft structures and thereby provides a reliable and reasonable clinical alternative for non-radiographic imaging in contrast to established radiological examination techniques.

### Supplementary Information

**Additional file 1.**

## Data Availability

All data generated or analyzed during this study are included in this published article [and its supplementary information files].

## Abbreviations

3 T- MRI: 3-Tesla magnetic resonance imaging
AI: Artificial intelligence
CBCT: Cone beam computed tomography
CT: Computed tomography
HASTE: Half-fourier acquisition single-shot turbo spin-echo
PETRA: Pointwise encoding time reduction with radial acquisition
UKE: Universitätsklinikum hamburg-eppendorf
VIBE: Volumetric interpolated brain examination

## References

[1] Paolo Maridati ES (2014). Stefano Speroni, Marco Cicciu, Carlo Maiorana: alveolar antral artery isolation during sinus lift procedure with the double window technique. Open Dent J.

[2] RAJ PJB. Grafting of the maxillary sinus floor with autogenous marrow and bone. J oral Surg. 1980;38. 6993637

[3] Andrés-García RR-SJ, Herrero-Climent M, Bullón P, Fernández-Farhall J, Gómez-Menchero A, Fernández-Palacín A, Ríos-Carrasco B. Sinus floor elevation via an Osteotome technique without biomaterials. Int J Environ Res Public Health. 2021;27. 10.3390/ijerph18031103 PMC7908564 33513756

[4] Laurino FA, Choi IG, Kim JH, Gialain IO, Ferraço R, Haetinger RG, Pinhata-Baptista OH, Abdala-Junior R, Costa C, Cortes AR. Correlation between magnetic resonance imaging and cone-beam computed tomography for maxillary sinus graft assessment. Imaging Sci Dent. 2020;50. 10.5624/isd.2020.50.2.93 PMC7314607 32601583

[5] Smeets RSM, Gauer T, Aarabi G, Assaf AT, Rendenbach C, et al. Artefacts in multimodal imaging of titanium, zirconium and binary titanium–zirconium alloy dental implants: an in vitro study. Dentomaxillofac Radiol. 2017;46. 10.1259/dmfr.20160267 PMC5595012 27910719

[6] Flügge TLU, Amrein P, Kernen F, Vach K, Maier J, et al. MRI for the display of autologous onlay bone grafts during early healing—an experimental study. Dentomaxillofac Radiol. 2020;50. 10.1259/dmfr.20200068 PMC7860956 33201739

[7] Nakamura T. Dental MRI: a road beyond CBCT. Eur Radiol. 2020;30. 10.1007/s00330-020-07321-7 32997171

[8] Mortazavi SMNM, Anoosheh SM, Bahaeddini N, Mortazavi G, Neghab P, Rajaeifard A. High-field MRI and mercury release from dental amalgam fillings. Int J Occup Environ Med. 2014;5. PMC7767616 24748001

[9] Hasegawa MMK, Abe Y, Ishigami T. Radiofrequency heating of metallic dental devices during 3.0 T MRI. Dentomaxillofac Radiol. 2013;42. 10.1259/dmfr.20120234 PMC3635774 23520391

[10] Khan MHHX, Tian X, Ouyang C, Wang D, Feng S, Chen J, Xue T, Bao J, Zhang X. Short- and long-term effects of 3.5-23.0 tesla ultra-high magnetic fields on mice behaviour. Eur Radiol. 2022;32. 10.1007/s00330-022-08677-8 35294587

[11] van Minde DKL, Weda H. Pinpointing moments of high anxiety during an MRI examination. Int J Behav Med. 2014;21. 10.1007/s12529-013-9339-5 24043600

[12] Korn P, Elschner C, Schulz MC, Range U, Mai R, Scheler U (2015). MRI and dental implantology: two which do not exclude each other. Biomaterials.

[13] Flügge T, Ludwig U, Winter G, Amrein P, Kernen F, Nelson K. Fully guided implant surgery using magnetic resonance imaging - an in vitro study on accuracy in human mandibles. Clin Oral Implants Res. 2020;31. 10.1111/clr.13622 32459868

[14] Niraj LK, Patthi B, Singla A, Gupta R, Ali I, Dhama K, Kumar JK, Prasad M (2016). MRI in dentistry- a future towards radiation free imaging -systematic review. J Clin Diagn Res.

[15] de Carvalho e Silva Fuglsig J, Wenzel A, Hansen B, Lund TE, Spin-Neto R. Magnetic resonance imaging for the planning, execution, and follow-up of implant-based Oral rehabilitation: systematic review. Int J Oral Maxillofac Implants. 2021;36. 10.11607/jomi.8536 34115055

[16] Reda RZA, Mazzoni A, Cicconetti A, Testarelli L, Di Nardo D. An update of the possible applications of magnetic resonance imaging (MRI) in dentistry: a literature review. J Imaging. 2021;7. 10.3390/jimaging7050075 PMC8321370 34460671

[17] Hilgenfeld TJA, Jende JME, Rammelsberg P, Heiland S, Bendszus M, Schwindling FS. Use of dental MRI for radiation-free guided dental implant planning: a prospective, in vivo study of accuracy and reliability. Eur Radiol. 2020;30. 10.1007/s00330-020-07262-1 PMC7599174 32960331

[18] Wanner L, Ludwig U, Hövener J, Nelson K, Flügge T. Magnetic resonance imaging (MRI) – a diagnostic tool for postoperative evaluation of dental implants. A case report. Oral Surg Oral Med Oral Pathol Oral Radiol. 2018;125. 10.1016/j.oooo.2018.01.005 29501353

[19] Gray CFRT, Smith FW, Staff R T (2003). Advanced imaging: magnetic resonance imaging in implant dentistry. Clin Oral Impl Res.

[20] Kocasarac HD, Geha H, Gaalaas LR, Nixdorf DR. MRI for dental applications. Dent Clin N Am. 2018;62. 10.1016/j.cden.2018.03.006 29903562

[21] Boeddinghaus R, Whyte A (2017). Trends in maxillofacial imaging.

[22] Mangano FG, Luongo F, Picciocchi G, Mortellaro C, Park KB, Mangano C (2016). Soft tissue stability around single implants inserted to replace maxillary lateral incisors: a 3D evaluation. Int J Dent.

[23] Widek TGP, Merkens H, Boldt J, Petrovic A, Vallis J, Scheurer E. Dental age estimation: the chronology of mineralization and eruption of male third molars with 3T MRI. Forensic Sci Int. 2019;297. 10.1016/j.forsciint.2019.02.019 30831415

[24] Bohner LTP, Meier N, Gremse F, Kleinheinz J, Hanisch M. Trabecular bone assessment using magnetic-resonance imaging: a pilot study. Int J Environ Res Public Health. 2020;17. 10.3390/ijerph17249282 PMC7763832 33322479

[25] Hovener JB, Zwick S, Leupold J, Eisenbeibeta AK, Scheifele C, Schellenberger F, Hennig J, Elverfeldt DV, Ludwig U (2012). Dental MRI: imaging of soft and solid components without ionizing radiation. J Magn Reson Imaging.

[26] Modolo JTA, Legros A (2017). Human exposure to power frequency magnetic fields up to 7.6 mT: an integrated EEG/fMRI study. Bioelectromagnetics.

[27] Al-Haj Husain ASM, Stadlinger B, Pejicic R, Winklhofer S, Piccirelli M, Valdec S (2021). Visualization of the inferior alveolar nerve and lingual nerve using MRI in Oral and maxillofacial surgery: a systematic review. Diagnostics (Basel).

[28] Schwindling FSBS, Herpel C, Kronsteiner D, Vogel L, Juerchott A, Heiland S, Bendszus M, Rammelsberg P, Hilgenfeld T. Geometric reproducibility of three-dimensional Oral implant planning based on magnetic resonance imaging and cone-beam computed tomography. J Clin Med. 2021;10. 10.3390/jcm10235546 PMC8658654 34884244

[29] Nagarajan APR, Thyagarajan R, Namasivayam A. Diagnostic imaging for dental implant therapy. J Clin Imaging Sci. 2014;4. 10.4103/2156-7514.143440 PMC4220422 25379354

[30] Geibel MA, Gelißen B, Bracher AK, et al. Artifact properties of dental ceramic and titanium implants in MRI. Exp Radiol Thieme. 2019;191. 10.1055/a-0755-2374 30419571

[31] Bohner L, Meier N, Gremse F, Tortamano P, Kleinheinz J, Hanisch M. Magnetic resonance imaging artifacts produced by dental implants with different geometries. Dentomaxillofac Radiol. 2020;49. 10.1259/dmfr.20200121 PMC7719859 32589480

[32] Duttenhoefer F, Mertens ME, Vizkelety J, Gremse F, Stadelmann VA, Sauerbier S (2015). Magnetic resonance imaging in zirconia-based dental implantology. Clin Oral Implants Res.

[33] Assaf ATZT, Remus CC, Schönfeld M, Habermann CR, Riecke B, Friedrich RE, Fiehler J, Heiland M, Sedlacik J. Evaluation of four different optimized magnetic-resonance-imaging sequences for visualization of dental and maxillo-mandibular structures at 3 T. J Cranio-Maxillofac Surg. 2014;42. 10.1016/j.jcms.2014.03.026 24837485

[34] Hilgenfeld TPM, Schwindling FS, Heil A, Kuchenbecker S, Rammelsberg P, Bendszus M, Heiland S. Artefacts of implant-supported single crowns - impact of material composition on artefact volume on dental MRI. Eur J Oral Implantol. 2016;9. 27722227

[35] Aiswarya Ashok PA, Devarajan E, Thavakkara V, Latha N, Saraswathy A. The effect of metallic dental restorations and implants in causing patient discomfort and artefacts during magnetic resonance imaging of the head and neck. Indian J Dent Res. 2022;33. 10.4103/ijdr.ijdr_430_21 36656184

[36] Bohner LHM, Sesma N, Blanck-Lubarsch M, Kleinheinz J. Artifacts in magnetic resonance imaging caused by dental materials: a systematic review. Dentomaxillofac Radiol. 2022;51. 10.1259/dmfr.20210450 PMC10043623 35348371

[37] Schulze RS, Berndt D, d'Hoedt B. On cone-beam computed tomography artifacts induced by titanium implants. Clin Oral Implants Res. 2010;21. 10.1111/j.1600-0501.2009.01817.x 19845706

[38] Georg Eggers MR, Kress B, Fiebach J, Dickhaus H, Hassfeld S. Artefacts in magnetic resonance imaging caused by dental material. Magma. 2005;18. 10.1007/s10334-005-0101-0 15785943

[39] Bohner LHM, Parize H, Sesma N, Kleinheinz J, Meier N. SEMAC + VAT for suppression of artifacts induced by dental-implant-supported restorations in magnetic resonance imaging. J Clin Med. 2023;12. 10.3390/jcm12031117 PMC9917855 36769765

[40] Bennardo FBS, Buffone C, Colangeli W, Antonelli A, Giudice A. Removal of dental implants displaced into the maxillary sinus: a retrospective single-center study. Head Face Med. 2022;17. 10.1186/s13005-022-00339-w PMC9670493 36397046

[41] Schorn LFT, Berndsen K, Kübler NR, Holtmann H, Rothamel D. The use of solvent-preserved human and bovine cancellous bone blocks for lateral defect augmentation - an experimental controlled study in vivo. Head Face Med. 2021;29. 10.1186/s13005-021-00275-1 PMC8240406 34187496

[42] Ali B, Syed M, Adam C, Zoga MD. Artificial intelligence in radiology: current technology and future directions. Semin Musculoskelet Radiol. 2018;22. 10.1055/s-0038-1673383 30399618

