# Supplementary material for: Assessment of the intrasinusidal volume before and after maxillary sinus augmentation using mri – a pilot study of eight patients
Source: BMC Oral Health. 2024 Jan 29;24:142. doi: 10.1186/s12903-024-03858-x (PMC10823631; doi:10.1186/s12903-024-03858-x)
Supplement: Supplementary file 1 — Additional file 1. [file 12903_2024_3858_MOESM1_ESM.docx]

**Appendix**

1. **Patient questionnaire**

Brief questionnaire of patient exposure to the research project:

„Assessment of bone by MRI before and after sinus augmentation".

Patient number: Date:

Dear Patient, please let us know by answering the following questions to what extent the magnetic resonance imaging (MRI) examination has bothered you.

Were you anxious before this MRI examination?

(1) Not at all

(2) Not really

(3) A little

(4) Very much

How did you perceive the preparation for this MRI examination?

As:

(1) Not at all unpleasant

(2) A little unpleasant

(3) Unpleasant

(4) Very unpleasant

Were you afraid during this MRI examination?

(1) Not afraid at all

(2) Not very afraid

(3) Afraid

(4) Very much afraid

Was this MRI examination painful?

(1) Not painful at all

(2) A little painful

(3) Painful

(4) Very painful

What was your overall perception of this MRI exam?

(1) Not at all unpleasant

(2) A little unpleasant

(3) Unpleasant

(4) Very unpleasant

How did you feel immediately after this examination?

(1) Very well

(2) Quite well

(3) Not so good

(4) Not good at all

**Please answer the following two questions only after the second MRI examination has been performed.**

How do you feel now (at the time of the second MRI examination)?

(1) Very well

(2) Quite well

(3) Not so good

(4) Not at all well

Has your perception of an MRI exam changed from the first MRI exam to the current MRI exam?

(1) Better from time to time

(2) Worse from time to time

(3) Same

(4) I cannot say

Thank you for your cooperation in this study!

1. **Alveolar nerve**


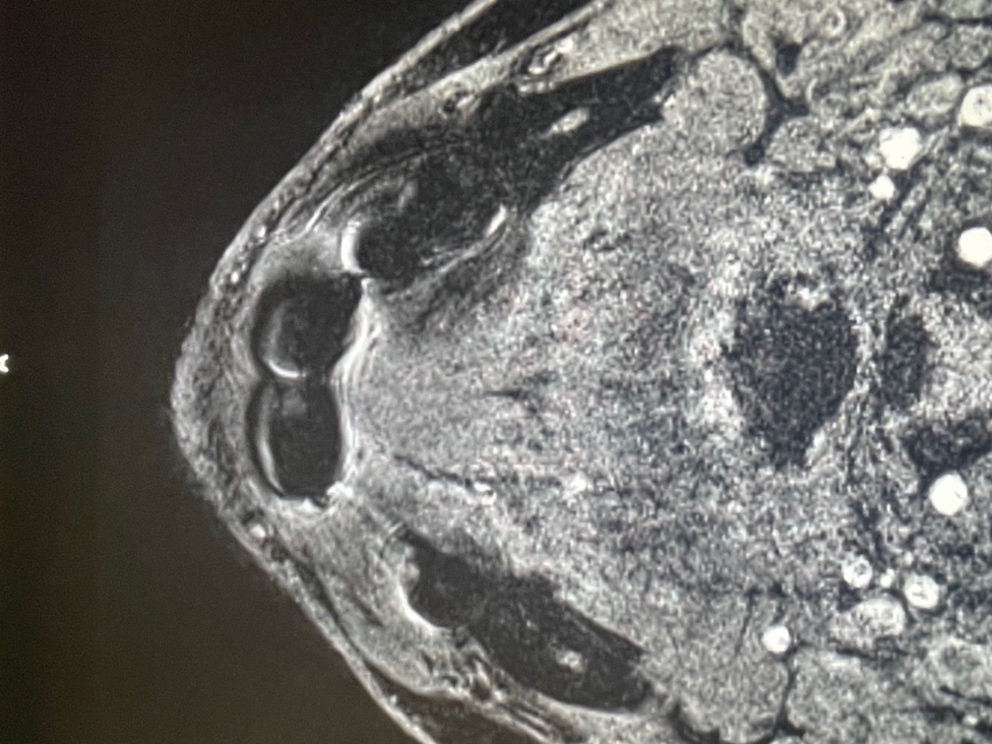


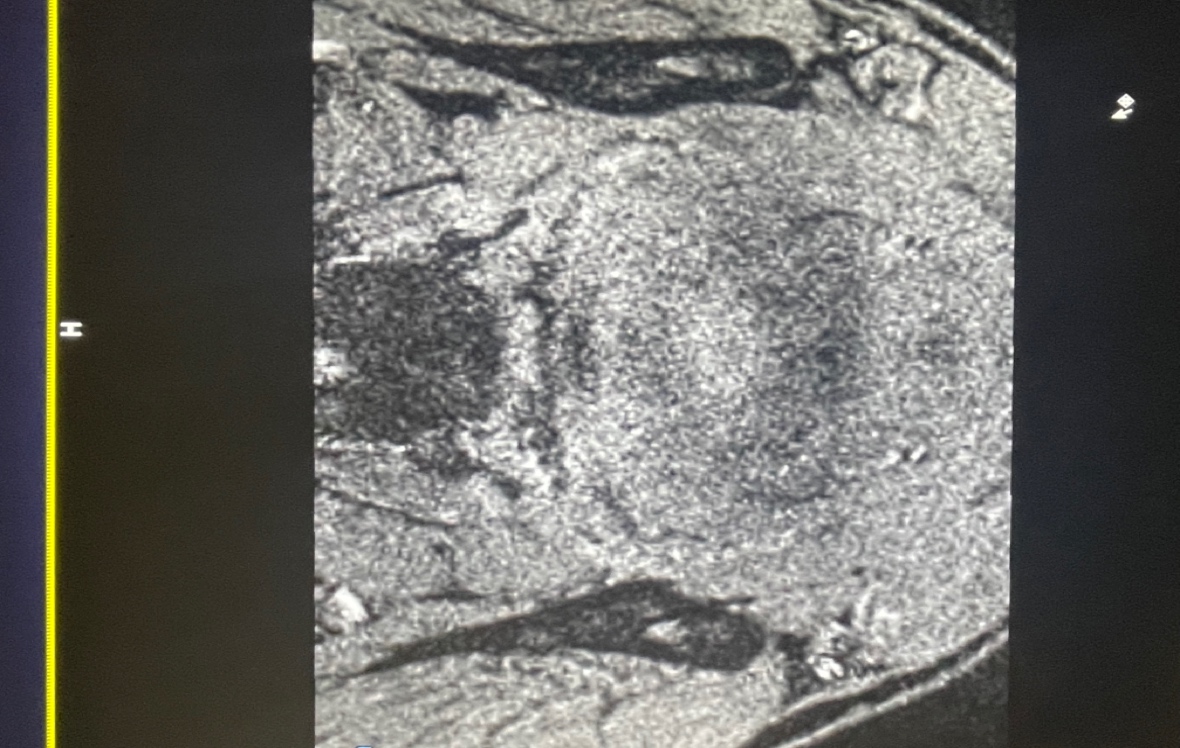


*Isotropic T1 weighted VIBE (volumetric interpolated brain examination) sequence in axial and coronal layer showing the inferior alveolar nerve (marked by arrow).*

1. **Examples for artefacts in MRI in this study**


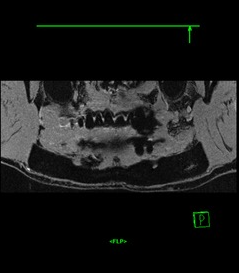


*Isotropic T1 weighted VIBE (volumetric interpolated brain examination) sequence, coronal layer showing focal magnetic susceptibility artifacts in the maxillary and mandibular region due to the distortions and signal change: local magnetic field inhomogeneities from metal restorations.*


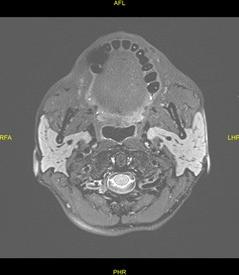


*Pd space (spc) fat saturated, axial layer showing focal magnetic susceptibility artifacts in the maxillary region due to the distortions and signal change: local magnetic field inhomogeneities from metal restorations.*


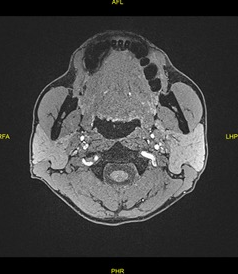


*Isotropic T1 weighted VIBE, axial layer showing focal magnetic susceptibility artifacts in the mandibular region due to the distortions and signal change: local magnetic field inhomogeneities from metal restorations.*
